# MiR 3180-5p promotes proliferation in human bladder smooth muscle cell by targeting PODN under hydrodynamic pressure

**DOI:** 10.1038/srep33042

**Published:** 2016-09-09

**Authors:** Yi Sun, De-Yi Luo, Yu-Chun Zhu, Liang Zhou, Tong-Xin Yang, Cai Tang, Hong Shen, Kun-Jie Wang

**Affiliations:** 1 Department of Urology, West China Hospital, Sichuan University, Chengdu, Sichuan, P. R. China; 2 Institute of Urology (Laboratory of Reconstructive Urology), West China Hospital, Sichuan University, Chengdu, Sichuan, P. R. China

## Abstract

Human bladder smooth muscle cells (HBSMCs) were subjected to pressure cycles of up to 200 cm H_2_O to a pressure of 0 cm H_2_O for 24 hours. The total RNA extracted from each group was subjected to microarray analysis. miR-3180-5p emerged as the most overexpressed of all the differentially expressed microRNAs, and this finding was validated by PCR. We then used CCK-8 to quantify cell proliferation after liposome-mediated transfection. Subsequently, we investigated the change in PODN and its downstream signaling proteins, including cyclin-dependent kinase 2 (cdk2) and p21. In addition, flow cytometry was performed to quantify cell-cycle distribution. The results show that miR-3180-5p, the microRNA that was most overexpressed in response to HP, reduced the expression of PODN and podocan (p = 0.004 and p = 0.041, respectively). Silencing of PODN via miR-3180-5p overexpression revealed a significant promotion of cell proliferation increased in the CCK-8 experiment, p = 0.00077). This cell proliferation was accompanied by an increase in cdk2 expression (p = 0.00193) and a decrease in p21 expression (p = 0.0095). The percentage of cells in (S + G2/M) improved after transfection (p = 0.002). It was apparent that HP upregulates miR-3180-5p, which inhibits the expression of PODN and promotes HBSMC proliferation via the cdk2 signaling pathway.

Mechanical stimuli are the key regulators of cell structure and function in both normal and diseased conditions^1^. A healthy bladder is exposed to cyclic variations in hydrostatic pressure during the normal course of urine filling and emptying^2^. Bladder smooth muscle cells (BSMCs), a major constituent of the bladder wall, represent the main cell type that is exposed to hydrostatic pressure (HP). Furthermore, bladder voiding cycles are regulated by human BSMCs (HBSMCs), which are the predominant group of cells that sense mechanical stimuli (viz., hydrostatic pressure on the bladder) and that translate the stimuli into molecular signals by altering the expression of proteins such as integrins, mitogen-activated protein kinases 1/2, and PI3K/SGK1 in order to alter the contractile force of the entire bladder^3^,^4^,^5^. Although the molecules involved in such regulation of bladder responses are known, the detailed mechanisms that translate mechanical stimuli into contractile responses remain unclear.

MicroRNAs are evolutionarily conserved, small noncoding RNAs that regulate approximately 30% of protein-coding gene expression at the post-transcriptional level. Hence, they regulate a wide variety of biological processes, including cell differentiation, growth, and apoptosis^6^,^7^,^8^,^9^. Increasing evidence suggests that microRNAs are involved in the proliferation of smooth muscle cells (SMCs) (e.g., vascular and airway SMCs)^10^,^11^. However, the role of microRNAs in the regulation of HBSMC proliferation in conditions other than bladder carcinomas remains poorly understood^12^. Furthermore, the induction of specific microRNAs in response to hydrostatic pressure remains unknown.

In the present study, we investigated whether hydrostatic pressure can induce microRNA expression and whether differentially expressed microRNAs are involved in the regulation of BSMC proliferation. Furthermore, we attempted to examine the molecular mechanisms by which such microRNAs may exert their regulatory effect.

## Results

### Culture and identification of HBSMCs

**Figure 1:**
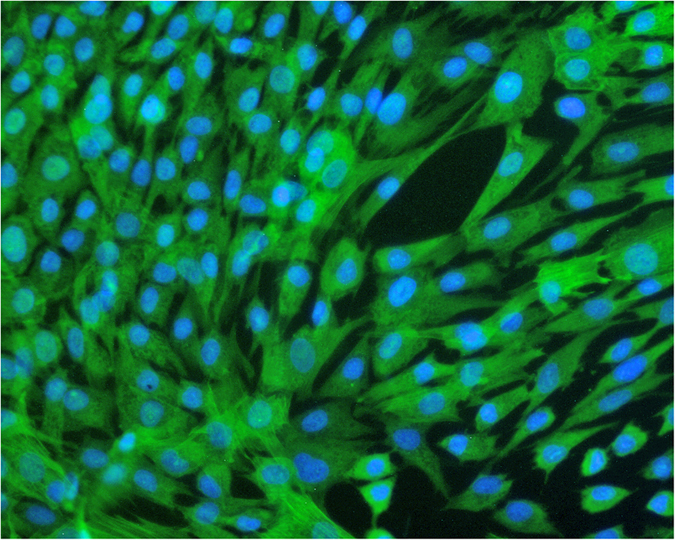
Immunofluorescence staining showing the condition of HBSMCs. Experiments were performed using cells between passages 3 and 5 that exhibited normal morphology and good activity.

Before the experiment, we used immunofluorescence to measure the condition of the cells. More than 95% of the cells showed positive immunofluorescent staining for the SM cell marker α-SM actin (Fig. 1).

### Altered microRNA expression in response to hydrostatic pressure

**Figure 2:**
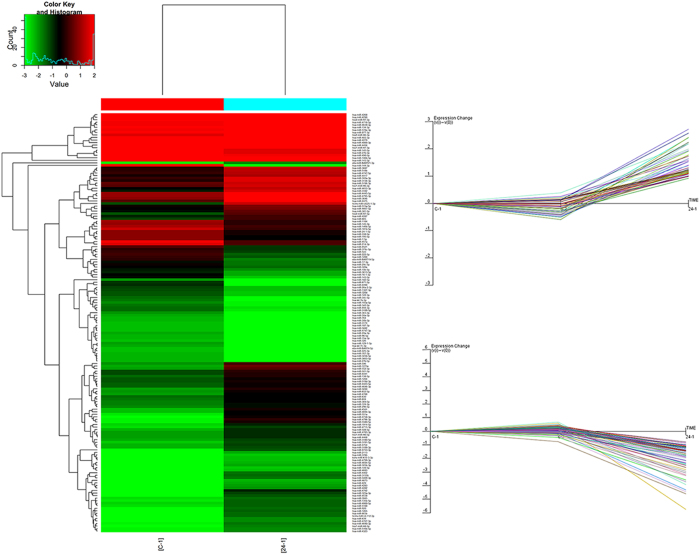
Hierarchical clustering and stem analysis of the microRNAs. The heat map diagram shows the result of the two-way hierarchical clustering of miRNAs and samples. Each row represents an miRNA, and each column represents a sample. The miRNA-clustering tree is shown on the left, and the sample-clustering tree appears at the top. The color scale shown at the top illustrates the relative expression level of an miRNA in the certain slide: red represents high relative expression levels, and green represents low relative expression levels. To analyzethe different expressions of microRNAs according tothe stem analysis, all of the altered microRNAs have continually changing trends in the stem analysis.

The total RNA isolated from HBSMCs 24 h after the application of hydrostatic pressure was used for microarray and data analyses, which revealed the differential expression of specific microRNAs between the cells exposed to hydrostatic pressure and control cells. The HBSMCs contained 103 upregulated microRNAs and 60 downregulated microRNAs in response to hydrostatic pressure (Fig. 2). After target forecasting and functional analysis of these differentially regulated microRNAs, nine upregulated and four downregulated microRNAs were found to be related to cell proliferation. The upregulated microRNAs included miR-3180-5p, miR-3189-3p, miR-369-5p, miR-4283, miR-4287, miR-4323, miR-483-3p, miR-501-5p, and miR-552-3p (Table 1), and the downregulated microRNAs included miR-106b-5p, miR-17-3p, miR-374c-5p, and miR-543 (Table 1).

### Validation of microarray results by RT-PCR

**Figure 3:**
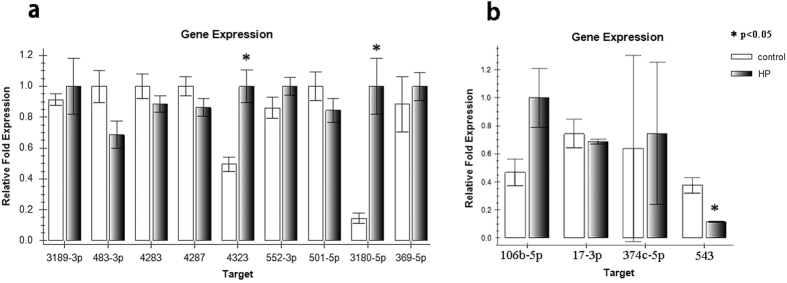
Different microRNA expressions were validated by PCR in the HBSMCs between the hydrostatic pressure (HP) and the control static group. (**a**) Of all the microRNAs, only miR 3180-5p and miR 4323 were significantly increased (p = 0.043, p = 0.027); (**b**) only miR 543 was significantly decreased (p = 0.048).

Microarray results were validated by RT-PCR. MiR-3180-5p and miR-4323 were significantly upregulated (p = 0.043 and p = 0.027, respectively; Fig. 3a), whereas miR-543 was significantly downregulated (p = 0.048; Fig. 3b). In addition, miR-3180-5p was the microRNA most upregulated in response to hydrostatic pressure (Fig. 3a); thus, it was selected for further validation experiments. This finding indicated that miR-3180-5p was likely involved in the process of HBSMC proliferation.

### Hydrostatic pressure-induced miR-3180-5p participates in proliferation

**Figure 4:**
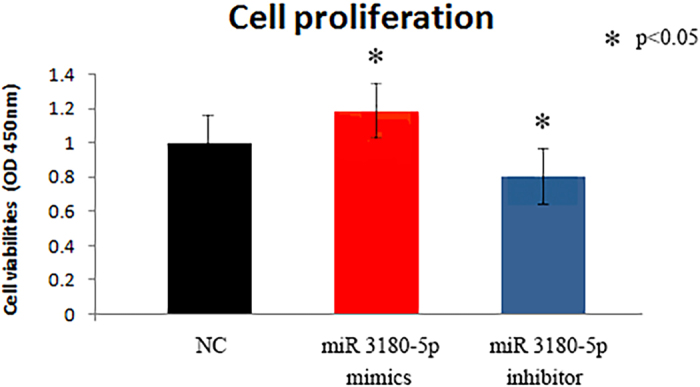
The proliferations of HBSMCs after overexpression or suppression of miR 3180-5p. The CCK-8 results demonstrated that miRNA 3180-5p regulates cell proliferation. Cell viability were markedly increased in the mimics group (p = 0.00077) and markedly attenuated in the inhibitor group (p < 0.00001).

We focused on miR-3180-5p because it was the most upregulated by hydrostatic pressure. Hydrostatic pressure is known to increase HBSMC proliferation^3^,^4^,^5^. Hence, we tested cell proliferation via a CCK-8 experiment after transfecting HBSMCs with either miR-3180-5p mimics or a miR-3180-5p inhibitor, which increased and decreased the intracellular levels of microRNA, respectively. The cell viability was markedly increased in the mimics group (p = 0.00077) and markedly attenuated in the inhibitor group (p < 0.00001). Thus, miR-3180-5p regulates HBSMC proliferation under hydrostatic pressure. All of these results are presented in Fig. 4.

### PODN expression was suppressed by miR-3180-5p

**Figure 5:**
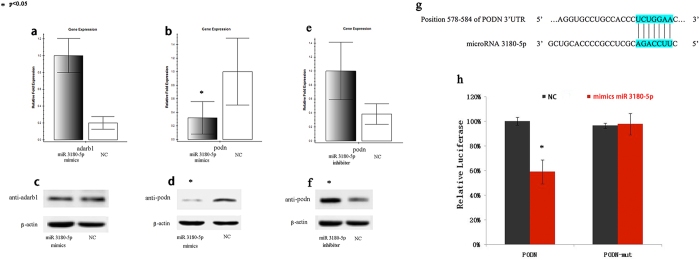
The miRNA 3180-5p regulates the expression of PODN. After upregulation of miR 3180-5p by transfection, (**a**) the mRNA level of ADARB1was increased (p = 0.008), and (**b**) the mRNA level of PODN was markedly decreased (p = 0.001). (**c**) Adenosine deaminase RNA-specific B1, the functional protein of ADARB1, had no change (p = 0.134). (**d**) The protein level of PODN was markedly decreased in the miR 3180-5p mimics-treated cells. After downregulation of miR 3180-5p via transfection, (**e**) the mRNA level of PODN was markedly increased (p = 0.0035), and (**f**) the protein level of PODN was markedly increased (p = 0.025). (**g**,**h**) T293T cells were co-transfected by psiCHECK-PODN (wild or mutant type) with mimics miR. The results showthat upon transfection with wild-type psiCHECK-PODN reporter vectors, luciferase activity was decreased by ~41% (p = 0.0022); moreover, mutation of the binding site markedly suppressed the effect of miR 3180-5p (p = 0.8127).

Previous research revealed that miR-3180-5p is involved in HBSMC proliferation. The expression levels of ADARB1 and PODN in the HBSMCs were validated by RT-PCR and western blot to investigate the effect of the overexpression of miR-3180-5p on target mRNA. In cells transfected with miR-3180-5p mimics, the mRNA level of PODN was found to be decreased (p = 0.004), whereas that of ADARB1 was found to be increased (p = 0.008). We corroborated this result at the protein level via western blot. Our study revealed that the expression of podocan, the functional protein product of the PODN gene, could be suppressed by miR-3180-5p overexpression in HBSMCs (p = 0.041). However, adenosine deaminase RNA-specific B1, the functional protein of ADARB1, showed no changes (p = 0.134) in response to miR-3180-5p. Moreover, the levels of PODN and podocan were demonstrably increased in cells transfected with the miR-3180-5p inhibitor (p = 0.0035; p = 0.025). All of these results are shown in Fig. 5(a–f).

### PODN was directly inhibited by miR-3180-5p

Computational prediction of human microRNA targets using miRanda and TargetScan indicated that PODN is a potential target of miR-3180-5p. To establish the direct effect of mi- 3180-5p on the 3′ UTR of PODN, a dual luciferase assay was performed. Luciferase activity was decreased by ~41% (p = 0.0022) when wild-type psiCHECK-PODN reporter vectors were co-transfected with miR-3180-5p in 293T cells. In contrast, the luciferase activity of 293T cells transfected with mutant psiCHECK-PODN showed no significant difference from the NC group (p = 0.8127; Fig. 5g,h).

### Expression of cdk2 is promoted by miR-3180-5p

**Figure 6:**
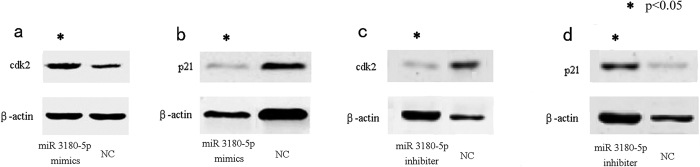
The expression of downstream proteins (cdk 2 signaling pathway). (**a**) The decrease in the expression of podocan was associated with an increase in cdk2 expression (p = 0.002) (**b**) and a decrease in p21 expression (p = 0.0095). (**c**) The increase in expression of podocan was associated with a decrease in cdk2 expression (p = 0.0002) (**d**) and increase in p21 expression (p = 0.011).

To further validate the inhibitory effect of miR-3180-5p on podocan expression, we investigated the effect of miR-3180-5p on the proteins p21 and cdk2, which function downstream of podocan. Figure 6 shows that decreased expression of podocan was associated with an increase in cdk2 expression (Fig. 6a, p = 0.002) and a decrease in p21 expression (Fig. 6b, p = 0.0095), while increased expression of podocan was associated with a decrease in cdk2 expression (Fig. 6c, p = 0.0002) and an increase in p21 expression (Fig. 6d, p = 0.011). The above results suggest that the function of cdk2 was affected by miR-3180-5p via the regulation of podocan.

### miR-3180-50 facilitated the G1 to S transition in the cell cycle

**Figure 7:**
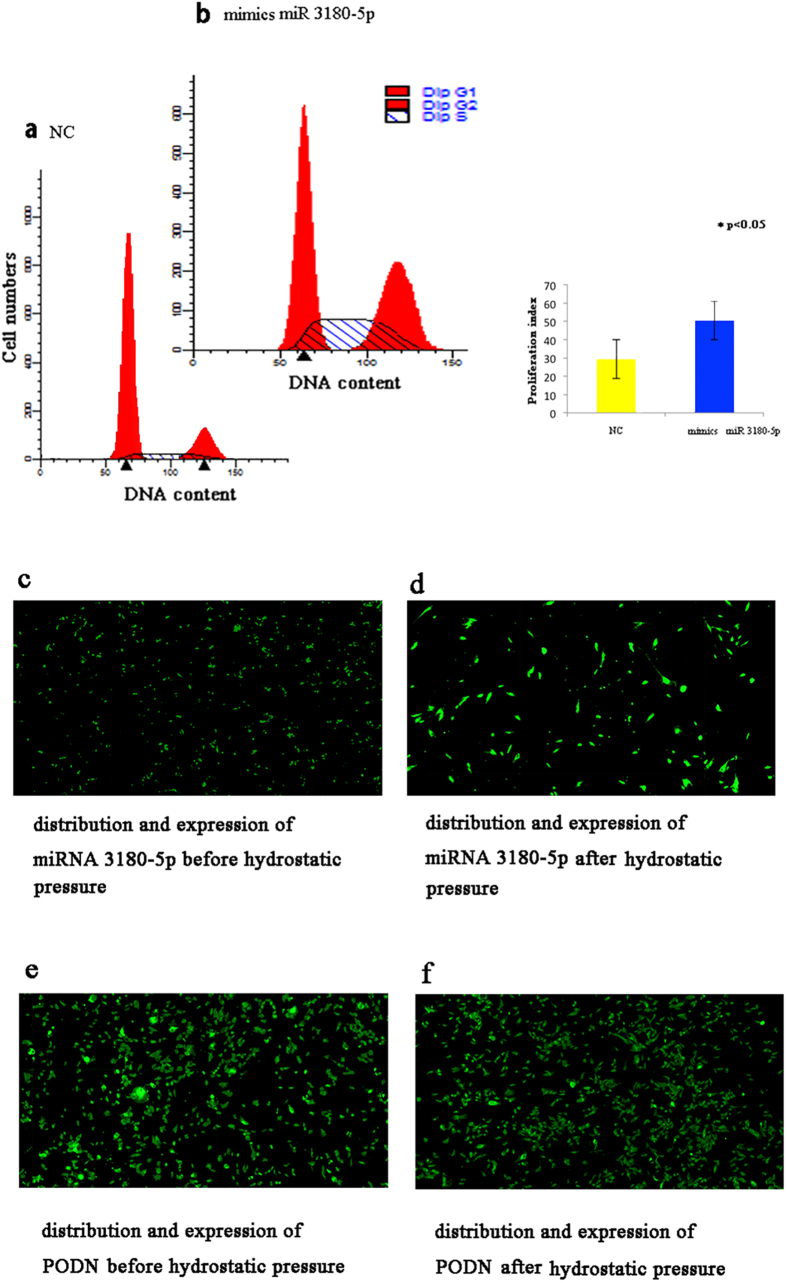
The effect of miRNA 3180-5p on the cell cycle. The expression of miRNA 3180-5p and PODN in patient samples. (**a**,**b**) The proliferation index, which is the percent of (S + G2/M), increased from 29.32 ± 6.25 in the NC group (non-targeting control sequence-treated cells) to 50.50 ± 3.41 in the miR 3180-5p mimics group (p = 0.002). The miR 3180-5p promoted cell transformation from the G1 to S phase. After induction of hydrostatic pressure, the expression of miRNA 3180-5p was up-regulated ((**c**,**d**) p < 0.00001), while the expression of PODN was down-regulated ((**e**,**f**) p < 0.00001).

After confirming the regulation of cdk2 by miR-3180-5p, flow cytometry was performed to test the effect of miR-3180-5p on the cell cycle by calculating the change in the percentage of cells in (S + G2/M). We transfected HBSMCs with miR-3180-5p mimics, and the increased intracellular levels of miR-3180-5p were accompanied by a significant increase (p = 0.002) in the proliferation index (the percentage of cells in (S + G2/M)) of HBSMCs, from 29.32 ± 6.25% in the NC group to 50.50 ± 3.34% in the cells transfected with miR-3180-5p (Fig. 7a,b). This result demonstrated that overexpression of miR-3180-5p, which is involved in the cell cycle, induced cells to progress from G1 to S phase, thus promoting the proliferation of HBSMCs. This result is consistent with our previous study, which showed that application of hydrostatic pressure resulted in an increase in the cell proliferation index from 25.8 ± 7.1% under static conditions to 39.7 ± 4.2% under HP^3^,^4^.

### The expression of miR-3180-5p and PODN in patient cells

Next, the expression levels of miR-3180-5p and PODN were tested in patient cells. In samples from patient cells, expression of miR-3180-5p was upregulated under hydrostatic pressure (Fig. 7c,d; p < 0.00001), whereas that of PODN was downregulated (Fig. 7e,f; p < 0.00001). These results were consistent with a previous study, confirmed the clinical relevance of miR-3180-5p, and demonstrated that a better understanding of the relationship between miR-3180-5p and proliferative signaling pathways may provide important novel targets for engineering bladder tissue.

## Discussion

Increasing evidence has demonstrated that mechanical stimulation plays an important role in the biological function of the urinary system^1^,^3^,^4^,^5^. MicroRNAs have emerged as potent and plausible regulators of biological responses^6^,^7^,^8^,^9^. Furthermore, HBSMCs exposed to mechanical stimulation are known to proliferate, but the molecular mechanisms involved in this process are not clearly understood^10^,^13^,^14^. Hence, we investigated the potential role of microRNAs in the regulation of HBSMC proliferation. Overall, our results demonstrated that high hydrostatic pressure leads to an increase in the expression of miR-3180-5p, which promotes HBSMC proliferation by the suppression of PODN, thereby leading to the activation of the pro-proliferative cdk2 pathway. To our knowledge, the identification of miR-3180-5p as a mechano-sensitive microRNA in HBSMCs represents the first evidence linking microRNA to cell proliferation in the bladder, and miR-3180-5p may be an important target for treatment of bladder diseases.

In this study, we focused on the mechanical stimulation of HBSMCs, as would be the case *in vivo* during routine bladder filling and voiding cycles, and we observed the differential expression of a distinct group of microRNAs. Using a genome-wide microarray, we identified nine upregulated microRNAs and four downregulated microRNAs in HBSMCs in response to hydrostatic pressure, and these results were corroborated by RT-PCR. Further, we determined the functional importance of the microRNA most sensitive to hydrostatic pressure, miR-3180-5p. Transfection of HBSMCs with mimics of miR-3180-5p or with a miR-3180-5p inhibitor clearly demonstrated that miR-3180-5p significantly promoted the proliferation of HBSMCs under hydrostatic pressure.

It has been reported that microRNAs can bind to target sites with partial complementation at the 3′ UTR of mRNA and can interfere with mRNA stability or translational efficiency^15^. We selected podocan, a novel member of the family of small leucine-rich repeat proteins that is commonly found in the extracellular matrix^16^,^17^ because proteins in that family are highly effective and selective modulators of important cellular functions^18^,^19^,^20^, including proliferation. Our data indicated that miR-3180-5p directly affects PODN, as observed by decreased PODN mRNA expression levels using RT-PCR and reduced podocan levels in western blots from cells exposed to miR-3180-5p, and this effect was confirmed as a direct effect based on a luciferase reporter assay. Therefore, it was apparent that miR-3180-5p inhibits PODN gene expression, leading to decreased production of its functional protein, podocan. Consistent with our findings, Shimizu-Hirota *et al*. reported that podocan overexpression is associated with an increase in p21 expression and a decrease in cdk2 expression^21^. The activation of a cdk2/cyclin complex is involved in the transition from G1 to S phase in the cell cycle, and this complex can be inhibited by p21^22^. Our flow cytometry results confirmed that the percentage of cells in (S + G2/M) increased when podocan levels were reduced by the overexpression of miR-3180-5p. This result essentially suggests that when miR-3180-5p is overexpressed, transcription of PODN is limited, and the levels of podocan decrease. This leads to the activation of its downstream pathway, the cdk2 signaling pathway, which promotes cellular proliferation. In addition, the changes in PODN, podocan, cdk2 and p21 were confirmed by a complementary approach utilizing a miR-3180-5p inhibitor. In summary, a better understanding of the relationship between microRNAs and proliferative signaling pathways may provide important novel targets for engineering bladder tissue.

However, there remain some limitations of our study. This study was based on overexpression by transfection, which can induce thousand-fold microRNA overexpression but only 7-fold hydrodynamic pressure. However, when the results are compared with previously published reports of cell proliferation under HP, the increase in cell proliferation is similar to that observed in our miR-3180-5p overexpressing transfected cells^3^. Thus, we can conclude that miR-3180-5p is involved in the HP-induced HBSMC proliferation. The next important point here is that the factors regulating the miR-3180-5p levels remain unknown. A few studies have shown that long noncoding RNAs (lncRNAs) inhibit or promote microRNA expression during cell biological processes^23^,^24^. Our preliminary gene-chip results showed that there are some lncRNAs that can target miR-3180-5p. However, this possibility requires further investigation.

## Methods

### HBSMC culture

HBSMCs (Cat. No. 4310; ScienCell, USA) were cultured in Dulbecco’s modified Eagle’s medium supplemented with 10% fetal bovine serum, penicillin (100 U/ml), and streptomycin (100 μg/ml) at 37 °C in a humidified chamber with 5% CO_2_. The morphological characteristics of HBSMCs were verified based on the using an optical microscope. Immunofluorescence was used to assess the expression of α-SM actin, the smooth muscle cell marker. Then, experiments were performed using cells between passages 3 and 5 that exhibited normal morphology and good activity.

### Hydrostatic pressure

*Ex-situ* hydrostatic pressure was applied to HBSMCs using a custom-designed motorized pressure apparatus, as previously reported^25^. In brief, the entire system was placed in a CO_2_ incubator to ensure constant temperature, atmosphere, and humidity. The HBSMCs were then subjected to cyclic hydrodynamic pressure (HP), simulating the natural bladder cycle (2 h/cycle). In each cycle, the pressure was slowly increased from 0 cm H_2_O to 10 cm H_2_O in the first 1.45 hours, then rapidly from 10 cm H_2_O to 200 cm H_2_O in the next 0.15 h, with a final pressure drop to 0 cm H_2_O at the end of the 2-h cycle. Such 2-h cycles were repeated for up to 24 h for HBSMCs, and these cells were referred to as the HP cells. Control HBSMCs were maintained under static conditions (0 cm H_2_O) in the same atmospheric conditions with computerized pressure monitoring and regulation.

### MicroRNA and mRNA microarray

Total RNA was extracted from HP and control cells using TRIzol (Invitrogen, Carlsbad, CA, USA) and miRNeasy mini kits (QIAGEN) according to the manufacturer’s instructions. The analyses of the data were also performed in accordance with the manufacturer’s instructions. The microRNAs and mRNAs showing differential expression in the hydrostatic pressure group and the static control group were identified through fold-change filtering (fold change ≥2.0)^26^. Target analyses of microRNAs and mRNAs were performed using three tools: Microcosm, miRanda, and TargetScan^26^. Only those microRNAs and mRNAs for which the target relationship could be established were analyzed further. Gene function prediction was performed using Kyoto Encyclopedia of Genes and Genomes (KEGG) and Gene Ontology (GO) categories^26^. Only those microRNAs that targeted genes inhibiting cell proliferation were chosen for validation.

### Real-time PCR

#### microRNA

Total RNA was extracted using TRIzol, and cDNA was synthesized using an All-in-One^TM^ miRNA qRT-PCR Detection Kit (GeneCopoeia, Foster City, CA, USA) according to the manufacturer’s instructions. Total microRNA was quantified using a *Homo sapiens* snRNA U6 qPCR primer as an internal control. The PCR conditions were also programmed according to the manufacturer’s instructions for the All-in-One^TM^ miRNA qRT-PCR Detection Kit from GeneCopoeia. The sequences were synthesized by GeneCopoeia.

#### mRNA

Total RNA was extracted using TRIzol, and cDNA was synthesized with an iScript^TM^ cDNA Synthesis Kit (Bio-Rad, Richmond, CA, USA) using the housekeeping gene GAPDH as an internal control. The PCR cycles were run according to the instructions for SYBR Premix Ex Taq II (TAKARA, Dalian, Liaoning, China). The primer sequences were as follows: (i) ADARB1: 5′-GAAACTCCTGACAAGGCGGA-3′, (ii) NOX4: 5′-GCCAACGAAGGGGTTAAACA-3′, and (iii) PODN: 5′-GCAAAACAACCGCCTGACTT-3′.

These PCRs were performed using a Bio-Rad iQ5 thermal cycler (Hercules, CA, USA), and the quality of the resulting PCR products was monitored via a post-PCR melt curve analysis.

### Transfection experiment

To validate the functional significance of the identified microRNAs, we selected the microRNA 3180-5p (miR-3180-5p) because it was found to be upregulated in response to hydrostatic pressure. HBSMCs were transfected with miR-3180-5p mimics or inhibitor at 100 nM (GenePharma, Shanghai, China) using the transfection reagent Lipofectamine 3000 (Invitrogen, Carlsbad, CA, USA) according to the manufacturer’s instructions. The control HBSMCs were treated with 10 nM non-targeting control sequence (GenePharma, Shanghai, China). Functional assays (flow cytometry and Western blots) were performed 48 h after transfection.

### Cell Counting Kit 8

Cell proliferation was assessed using a Cell Counting Kit 8 (CCK-8). The WST-8, which produces water-soluble formazan dye upon bioreduction in the presence of an electron carrier, was included in this system. After transfection, the HBSMCs (5 × 10^3^ cells/100 μl/well) were seeded into a 96-well flat-bottomed plate with culture medium (DMEM with 10% FBS). A blank control was performed using DMEM containing 10% CCK-8. The cells with CCK-8 (10 μl/well) were incubated at 37 °C for 2 h. After cultivation, absorbances at 450 nm (calibrated wave) were measured using a microplate reader (BioTek, Montpelier, VT, USA).

### Western blot analysis

At 48 h after transfection, the cells were lysed, and proteins were extracted and pooled using a Nuclear and Cytoplasmic Extraction Kit (CWBIO, Beijing, China) according to the manufacturer’s instructions. The extracted proteins were separated by 10% sodium dodecyl sulfate-polyacrylamide gel electrophoresis and subjected to western blot analyses using anti-cyclin-dependent kinase (cdk) 2 and anti-p21 and β-actin antibodies (all at 1:500; Abcam, Cambridge, MA, USA). Reactive protein bands were detected by the chemiluminescence method (ECL Plus Western Blot Detection System; Amersham Biosciences, Foster City, CA, USA).

### Luciferase assay

Luciferase constructs were synthesized using ligating oligonucleotides containing wild-type or mutant target sites for miR-3180-5p and were inserted into a psiCHECK-2 vector (Promega, Madison, WI, USA). For the luciferase assay, 293T cells (human embryonic kidney cells) were co-transfected with wild-type or mutant psiCHECK-PODN reporter vectors and miR-3180-5p mimics or a non-targeting control mimic sequence (as the NC groups) using Lipofectamine 3000 (Invitrogen). Luciferase activity was measured 48 h post-transfection using a Dual-Luciferase Assay Kit (Promega, Madison, WI, USA).

### Flow cytometry

HBSMCs were transfected with miR-3180-5p mimics, a miR-3180-5p inhibitor or a non-targeting control sequence, grown for 48 h, and then washed three times in phosphate-buffered saline (PBS). The cells were centrifuged at 1000 g for 5 min to separate the cells. The supernatant was discarded, and the pellet was fixed in 75% chilled ethanol overnight at 4 °C. Centrifugation was performed again, as described above, and the pellet was washed three times with PBS and re-suspended in 400 ml PBS containing 50 mg/mL RNase A and propidium iodide for 30 min in the dark. After filtration, the cell cycle was examined by flow cytometry using an EPICS ELITE ESP flow cytometer (Beckman Coulter, Miami, FL, USA). The cell proliferation index was calculated using a parameter that reflects the cell proliferation rate, according to the following formula: proliferation index (%) = (S + G2/M)/(G0/G1 + S + G2/M) × 100.

### RNA *in situ* hybridization of miR-3180-5p and PODN

To study the clinical relevance of miR-3180-5p and its target gene PODN, the expression levels of miR-3180-5p and PODN were investigated in patient samples by *in situ* hybridization. After receiving ethical approval and acquiring informed consent from the patients (patients undergoing radical cystectomy for muscle-invasive bladder cancer), we collected the normal bladders. Then, we isolated tissues from the macroscopically normal areas of the bladders. To verify normality, marginal samples were transferred to the Pathology Department, West China Hospital. Each suitable full-thickness tissue was collected and washed three times with PBS. In addition, we removed the urothelial and serosal layers from the smooth muscle layer. The remaining muscle layer was sheared to a 1 × 1 × 1 cm in size and then expanded in a culture dish.

RNA *in situ* hybridization for miR-3180-5p and PODN was performed after HBSMCs were subjected to cyclic hydrodynamic pressure. In brief, diethylpyro-carbonate (DEPC) was used to fix the cells, followed by digestion, autofluorescence elimination, blocking of endogenous biotin and degeneration. Next, samples were hybridized overnight with miR-3180-5p probe or PODN probe at 28 °C or 34 °C, respectively. After washing, the samples were blocked with bovine serum albumin (BSA; Solarbio, Beijing, China), and 488-Avidin (1:400; Sigma-Aldrich, Saint Louis, MO, USA) was added. DAPI was used to dye the cell nuclei. Images were captured using an inverted fluorescent microscope (Nikon ECLIPSE 80i, Tokyo, Japan). Image-Pro Plus 6.0 software was utilized to select the same light green used as a unified standard for all positive staining, and then for the analysis of each photo on every positive cumulative optical density.

### Statistical Analysis

All data are presented as the mean ± standard deviation (SD) and were analyzed by Student’s unpaired t-tests or by analysis of variance (ANOVA), as appropriate. P values of ≤0.05 were considered to be statistically significant.

### Statement about the methods

We confirm that all methods were carried out in accordance with relevant guidelines and regulations. And we confirm that all experimental protocols were approved by Institute of Urology (Laboratory of Reconstructive Urology), West China Hospital, Sichuan University and Ethics Committee of Sichuan University. In addition, we confirming that informed consent was obtained from all subjects.

## Conclusion

To our knowledge, this is the first study reporting the involvement of microRNA in cyclic HP-stimulated proliferation of HBSMCs. In this study, we demonstrated that hydrostatic pressure upregulated/downregulated multiple microRNAs in HBSMCs and that the proliferation of HBSMCs could be altered by the overexpression of a single miRNA (miR-3180-5p), which reduces PODN expression, leading to cdk2-mediated proliferation of HBSMCs. Thus, miR-3180-5p may be a novel molecular target for the regeneration of cells in engineering bladder tissue.

## Additional Information

**How to cite this article**: Sun, Y. *et al*. MiR 3180-5p promotes proliferation in human bladder smooth muscle cell by targeting PODN under hydrodynamic pressure. *Sci. Rep.* **6**, 33042; doi: 10.1038/srep33042 (2016).

## Figures and Tables

**Table 1:** The changed microRNA, target mRNA and the function of mRNA.

Changed microRNA	Change of miRNA	Target gene	Function of mRNA
microRNA 3180-5p	Up regulate	ADARB1	Negative regulation of cell proliferation
		PODN	Negative regulation of cell proliferation
microRNA 3189-3p	Up regulate	CDK10	Negative regulation of cell proliferation
		LYN	Negative regulation of cell proliferation
		PDS5B	Negative regulation of cell proliferation
microRNA 369-5p	Up regulate	NOX4	Negative regulation of cell proliferation
microRNA 4283	Up regulate	ADARB1	Negative regulation of cell proliferation
		CDK10	Negative regulation of cell proliferation
		IGFBP3	Negative regulation of cell proliferation
		NOX	Negative regulation of cell proliferation
		SMARCB1	Negative regulation of cell proliferation
microRNA 4287	Up regulate	ADARB1 LEPREL1	Negative regulation of cell proliferation
		LYN	Negative regulation of cell proliferation
		NOX4	Negative regulation of cell proliferation
			Negative regulation of cell proliferation
microRNA 4323	Up regulate	LYN	Negative regulation of cell proliferation
microRNA 483-3p	Up regulate	CDK10	Negative regulation of cell proliferation
		PODN	Negative regulation of cell proliferation
		SMARCB1	Negative regulation of cell proliferation
microRNA 501-5p	Up regulate	ADARB1 BMP4	Negative regulation of cell proliferation
		PDS5B	Negative regulation of cell proliferation
microRNA 552-3p	Up regulate	PBRM1	Negative regulation of cell proliferation
microRNA 106b-5p	Down regulate	DERL2	Positive regulation of cell proliferation
		LAMP3	Regulation of cell proliferation
		MDM4	Regulation of cell proliferation
		CSGALNACT1	Regulation of cell proliferation
microRNA 17-3p	Down regulate	MDM4	Regulation of cell proliferation
microRNA 374c-5p	Down regulate	EMP1	Regulation of cell proliferation
		MDM4	Regulation of cell proliferation
microRNA 543	Down regulate	CDH13	Regulation of cell proliferation
		MDM4	Regulation of cell proliferation
		EMP1	Regulation of cell proliferation
